# How Dangerous Is Obesity? Issues in Measurement and Interpretation

**DOI:** 10.1111/padr.12015

**Published:** 2016-12-12

**Authors:** Andrew Stokes, Samuel H. Preston

the prevalence of obesity is rising in nearly all regions of the world (Finucane et al. 2011). In the United States, the adult prevalence of obesity increased from 15 percent in 1976–1980 to 31 percent in 1999–2000 and to 38 percent in 2013–2014 (Flegal et al. 1998; Ogden et al. 2015; Flegal et al. 2012). Although it is well known that obesity raises risks for a variety of diseases, including cardiovascular disease, diabetes, and cancer (Asia Pacific Cohort Studies Collaboration 2004; Bogers et al. 2007; Wilson et al. 2008; Wolin, Carson, and Colditz 2010; Narayan and Boyle 2007), much uncertainty remains regarding the effects of excess weight on an individual's risk of death. For example, two large studies estimated that relative to the normal weight category, overweight and moderate obesity were associated with statistically significant increases in the risks of dying (Global BMI Mortality Collaboration 2016; Whitlock et al. 2009). A recent meta‐analysis, however, estimated that these same categories were associated with reduced likelihood of death, an association that was significant for overweight but not for moderate obesity (Flegal et al. 2013).

Recognizing the potential effect of rising obesity on mortality levels in the United States, the 2015 Technical Advisory Panel to the Social Security Administration recommended modeling the impact of changing levels of obesity in its projections (Technical Panel on Assumptions and Methods 2015). However, uncertainty is compounded in estimates and projections of the impact of obesity on the health of populations because these estimates are highly sensitive to small differences in relative risks (Flegal et al. 2005). A recent review found that published estimates of the proportion of deaths attributable to obesity in the United States ranged from 3 to 15 percent (Flegal, Panagiotou, and Graubard 2015). The first value would imply a relatively minor role for obesity, while the second figure would suggest that obesity is among the leading causes of death in the United States. A study of the impact of overweight and obesity on international differences in life expectancy found that high levels of obesity in the US accounted for 55 percent of its life expectancy shortfall using one set of risk factors but only 30 percent using another set (Preston and Stokes 2011).

A major reason for the wide uncertainty in estimates is that nearly all studies of the association between obesity and mortality are observational and are subject to the typical biases of observational studies. In an attempt to adjust for these biases, studies often impose severe exclusion criteria, which may reduce bias but limit generalizability to the population at large. In this article, we describe and illustrate the effects of the major biases and threats to generalizability associated with observational studies of the mortality risks of obesity using nationally representative data from the United States.

## Biases in estimates of the mortality risks of obesity

We distinguish between biases that are expected to understate the mortality risks associated with obesity (designated with a “U”) and those that are expected to overstate the risks (designated with an “O”).

### Biases that may underestimate the mortality risk of obesity

#### U.1. Reverse causation

Confounding by illness, also known as reverse causation, refers to the possibility that weight is the consequence rather than the cause of illness. The particular concern is that weight loss is often a result of a disease and that the disease is associated with higher mortality (Willett, Dietz, and Golditz 1999; Willett et al. 2005). Diseases that often precipitate weight loss include chronic obstructive pulmonary disease, heart failure, and many cancers. The high mortality and disease prevalence among individuals who have lost significant weight have been demonstrated in several studies (Preston, Mehta, and Stokes 2013; Stokes and Preston 2016b; Ingram and Mussolino 2010). Illness‐induced weight loss is a serious obstacle to obtaining unbiased estimates of the association between obesity and mortality (Ferrucci and Alley 2007; Hu 2008; Lawlor et al. 2006; Manson et al. 1987; Manson et al. 2007; Metha 2015; Tobias and Hu 2013; Wannamethee, Shaper, and Walker 2001; Willett et al. 2005; Willett, Hu, and Thun 2015; Willett et al. 1999).

Researchers have employed several strategies to reduce the biases associated with reverse causation. One is to delay the beginning of analysis until several years after baseline because the impact of reverse causal processes that may be at work at the time of survey collection is thought to be most consequential in the early years after the survey (Mehta 2015; Hu 2008). Their impact may, of course, stretch for much longer periods (Alley et al. 2010). A second strategy is to exclude from analysis people with various chronic conditions at baseline (Hu 2008). This approach would fail to capture subclinical illnesses. Both strategies eliminate large proportions of observations, thus reducing statistical power and potentially compromising external validity (Flegal et al. 2007). A third strategy is to control statistically for the presence of various illnesses present at baseline. Controlling for the presence of illness, or eliminating people with illness, may reduce the effects of reverse causation, but it also risks understating the mortality risks of obesity because the conditions controlled or eliminated may be a product of obesity itself. This would be an example of an “overadjustment bias” that is described below (Schisterman, Cole, and Platt 2009).

#### U.2. Residual confounding by smoking status

Smoking is widespread, deadly, and inversely associated with obesity. This combination poses a significant threat to unbiased estimates of the mortality risks of obesity. A review of epidemiologic and biomedical studies of the effect of smoking on weight suggests that US smokers weigh, on average, 4–5 kg less than non‐smokers (Audrain‐McGovern and Benowitz 2011). Former smokers gain, on average, 4.5 kg within 6–12 months after quitting.

Some studies of the mortality risks associated with obesity ignore smoking altogether (Olshansky et al. 2005). However, most studies of the mortality risks of obesity adjust for smoking by employing two or three smoking categories. But smoking is a complex exposure with multiple dimensions of duration and intensity and is difficult to measure precisely in self‐reported data, as comparisons of self‐reports to serum cotinine levels make clear (Stram, Huberman, and Wu 2002). A modest amount of measurement error in smoking, combined with the observed inverse association between smoking and body mass index (BMI), produces a spurious negative relation between obesity and mortality (Renehan, Leitzmann, and Zwahlen 2012). A common strategy to deal with this problem is to confine analysis to individuals who have never smoked. As in the case of strategies for dealing with reverse causation, however, such exclusions may introduce problems of their own by impairing generalizability.

Biases U.1 and U.2 are expected to be more severe among individuals who are ill with some specified disease or condition. The mortality risks of obesity are sometimes investigated among such samples. These individuals are obviously sicker than average, enhancing the risk of reverse causation; and smokers are likely to be overrepresented in populations of sick individuals because smoking is associated with an increased incidence of many diseases. Furthermore, the inverse association between smoking and obesity is typically even stronger in diseased groups than in the general population, enhancing the potential for confounding. These biases play a key role in producing the so‐called obesity paradox—lower mortality of obese people compared to non‐obese—among people with diabetes and pre‐diabetes (Preston and Stokes 2014) and cardiovascular disease (Stokes and Preston 2015).

#### U.3. Narrow observational windows

One might analyze the mortality risks of smoking by comparing outcomes for individuals who had smoked in the past year to those who had not. But such a design ignores a great deal of relevant history. For example, former smokers have higher mortality than those who never smoked, especially if they have quit recently (Ben‐Shlomo et al. 1994). This result reflects the long‐lasting impact of smoking on health.

There is accumulating evidence that obesity also leaves an enduring imprint on subsequent mortality. When observations of body mass index at different stages of life are introduced into the research design, they typically have predictive value. Preston, Mehta, and Stokes (2013) use a multivariate framework and find that BMI values recorded at three life‐cycle stages contribute additively to predicting mortality. Several studies (Abdullah et al. 2011; Mehta et al. 2014; Preston, Mehta, and Stokes 2013; Bouchard et al. 2013) conclude that mortality is a positive function of the duration of obesity, a feature of an entire BMI history. One's maximum BMI, a summary measure of one's weight trajectory, has also been introduced into hazard models (Mehta et al. 2014; Stokes 2014). Stokes and Preston (2016b) use formal tests of model performance to compare models based on one's maximum BMI to those based on a single measure of BMI at baseline. Using the Akaike Information Criterion (AIC) and the Bayesian Information Criterion measures (BIC), they find that the performance of the maximum BMI model was decisively superior to the survey BMI model.

Despite evidence that the risks of obesity are cumulative, most studies of the mortality risks of obesity are based on a single static measure of exposure. Body mass index is recorded once, at the time of the survey, and individuals are followed forward from that point. Relative to the underlying relation that exists between BMI history and mortality, having only one observation can be thought of as introducing measurement error into the relation. Even if such measurement error were random, it would be expected to bias results toward the null, that is, to produce an underestimate of the risks of obesity when considered as an historical process (Carroll et al. 2006; Greene and Cai 2004).

#### U.4. Statistical control of variables on the causal pathway between obesity and death

Some studies of the association between obesity and mortality control for conditions whose incidence is influenced by obesity and whose presence elevates the risk of death. Such procedures are an instance of what has been termed overadjustment bias (Schisterman et al. 2009). Instances of this practice occur when the incidence or prevalence of hypertension, diabetes, or cardiovascular disease is controlled when studying the association between obesity and mortality (Willett, Dietz, and Colditz 1999). Because obesity raises the incidence and prevalence of diabetes and cardiovascular disease, controlling for these diseases eliminates prominent pathways through which obesity affects mortality and biases downward estimates of the total risk of obesity (Schisterman et al. 2009). Flegal et al. (2013) conclude that 34 out of 97 studies that they reviewed were “possibly over‐adjusted” in this fashion.

### Biases that may overestimate the mortality risk of obesity

#### O.1. Failure to control for socioeconomic variables

Some studies of the association between obesity and mortality, including two of the largest analyses of the relation between BMI and mortality (Whitlock et al. 2009; Aune et al. 2016), make no effort to control for socioeconomic variables such as educational attainment, income, or occupation. In high‐income countries, obese people are drawn disproportionately from lower socioeconomic groups. For example, among non‐Hispanic whites aged 45–64 in the US, 47.0 percent of women who did not complete high school were obese in 1999–2006 compared to 31.5 percent of women who completed college. Among men, the corresponding values were 42.0 and 28.5 percent (Yu 2012). An inverse relation has also been demonstrated between obesity and income (Lundborg, Nystedt, and Rooth 2014).

Individuals with lower educational attainment and income are subject to a variety of influences beyond obesity that raise mortality risks (Elo 2009; Marmot 2004). These include poorer access to health care, greater exposure to infectious disease, poorer diets, more dangerous neighborhoods, and more stressful occupations. When socioeconomic status is not included in the analysis, these associated hazards that are positively correlated with obesity inappropriately inflate the coefficient on obesity. On the other hand, bias due to inadequate control for socioeconomic status may operate in the reverse direction in some low‐ and middle‐income countries where obesity is positively associated with socioeconomic status (Dinsa et al. 2012).

#### O.2. Measurement error in weight and height

Measurement error in weight and height could either increase, decrease, or leave unaffected the estimated impact of obesity on mortality. If measurement error were random, it would be expected to bias results toward the null, that is, to produce an underestimate of the risks of obesity (Carroll et al. 2006; Greene and Cai 2004). But several studies show that error in self‐reported weight and height is not random but rather produces underestimates of BMI both because weight is, on average, underestimated and because height is, on average, overestimated (Stommel and Schoenborn 2009). These errors create inappropriate “migration” of individuals from their proper BMI category to a lower self‐reported BMI category, producing estimates of the mortality risks associated with obesity that are too high compared to results based on measured weight and height (Flegal et al. 2013; Flegal, Kit, and Graubard 2014; Preston, Fishman, and Stokes 2015). Regression‐based methods for correcting self‐reported weight and height have been proposed (Ezzati et al. 2006; Burkhauser and Cawley 2008).

We have demonstrated that errors in self‐reported weight and height at survey are relatively small in the National Health and Nutrition Examination Survey, resulting in a correlation of 0.95–0.96 between measured BMI and self‐reported BMI (Preston, Fishman, and Stokes 2015). However, larger problems arise when discrete categories are imposed on self‐reported data; 20 percent of individuals were reported to be in a different 5‐unit BMI category from their correct category. This pattern of misreporting produced a relative risk associated with being moderately obese (30.0<BMI<35.0) of 1.40 when self‐reports were used, compared to a value of 1.25 when measured values were used. When a continuous measure of BMI was used, the bias was very small: a 5‐unit increment in BMI above 25.0 was associated with a relative risk of 1.28 using measured data and a relative risk of 1.30 using self‐reports (Preston, Fishman, and Stokes 2015).

## Threats to generalizability

In order to reduce biases associated with reverse causality and residual confounding by smoking, analysts often estimate the mortality risks of obesity after excluding ever‐smokers and people with chronic conditions from the analysis (Allison et al. 1999; Calle et al. 2003; Mokdad et al. 2004). By restricting analysis to individuals who are unusually healthy, however, researchers reduce the number of risks that compete with obesity. This restriction can magnify the relative risks of obesity.

To illustrate, suppose that the risks of death associated with obesity are additive with those of smoking: being obese increases the death rate by the same absolute amount for smokers and non‐smokers (Stokes and Preston 2016a; Mehta and Preston 2016) Then the *relative* risk of death associated with obesity will be higher for non‐smokers, who have a lower base of mortality, than for smokers. Consistent with this reasoning, four large studies have found that the relative risk of death associated with obesity was greater among non‐smokers or never‐smokers than among current smokers or ever‐smokers (Calle et al. 1999; Koster and Leitzmann 2008; van Dam et al. 2008; Ma et al. 2013). Two of these studies tested for the significance of differences in the association between obesity and smoking and found the interaction to be significant.

To our knowledge, every study that reports the relation between obesity and mortality does so in terms of relative risks, expressing the ratio of mortality among the obese to that among people of normal weight. When this is the metric employed and risks are additive, the results for healthy groups will not be generalizable to the population as a whole.

This problem is pervasive in studies of the mortality risks of obesity. Few of the studies cited here are based on national probability samples. Many take advantage of convenience samples that were generated for other purposes, and these often maintain an overrepresentation of white‐collar groups with unusually low mortality. These include the two largest studies of the relations between obesity and mortality: 900,000 Americans in the Cancer Prevention Study II cohort (Calle et al. 1999) and 530,000 members of the NIH/AARP cohort (Adams et al. 2006). White, college‐educated individuals were heavily overrepresented in these cohorts.

Relations between obesity and age are another instance in which the relative mortality risks of obesity decline when other risks increase. Wang (2014) conducted a meta‐analysis of studies that investigate interactions between age and obesity in their association with mortality and concluded that the relative risk of death among the obese declines sharply with age. One explanation of this finding is that the mortality risks of obesity become overwhelmed as many age‐related diseases advance. Other explanations of the decline in the risks of obesity with age include a greater intensity of reverse causal processes as age advances.

## Illustrations

We illustrate these biases and threats to generalizability in a new set of estimates of the excess mortality associated with obesity. As is conventional in the literature, we express the risks associated with obesity in terms of hazard ratios for all‐cause mortality. We develop a preferred set of estimates based on the choices reflected below.

1) We address biases associated with reverse causation (U1) and narrow observational windows (U3) by using as our principal indicator of obesity the maximum BMI that an individual has attained in the course of life, rather than BMI at baseline. The relation between BMI and illness can be considered the joint outcome of two separate processes: one, the effect of obesity on an individual's health; and two, any weight loss that is attendant upon illness (Alley et al. 2010). Failure to account for the latter process would likely lead to an underestimate of the effect of obesity on health. Because the maximum weight that an individual has achieved is not as sensitive to illness‐induced weight loss, the use of maximum weight is expected to reveal more precisely the effect of obesity on health (Stokes 2014; Stokes and Preston 2016b). Furthermore, as a characteristic of an entire weight history, one's maximum BMI is likely to be more comprehensive than a snapshot taken at baseline. As such, maximum BMI should be better able to capture the persistent effect of obesity on subsequent mortality.

2) We address the bias produced by failing to control for socioeconomic status (O1) by controlling for educational attainment and race. In contrast to other potential measures of socioeconomic status such as occupation or income, educational attainment is typically available for everyone. And since individuals complete their education in young adulthood, it is much less vulnerable to reverse causal pathways (i.e., health impairments affecting status) than are occupation or income (Elo 2009).

3) We address residual confounding by smoking (U2) by using detailed categories to control for smoking status. These categories are described below. Comparing results of models in which smoking is controlled to those where it is uncontrolled illustrates the importance of smoking for estimating the mortality hazards associated with obesity. However, residual confounding by smoking is still likely to be present in our results when smoking is controlled because there will still be measurement error in the smoking variables. Therefore, we present a model that is estimated on a data set that includes only individuals who have never smoked, even though such a restriction impairs the generalizability of results.

4) The remaining bias is that associated with measurement error in self‐reports of weight and height (O2). We partially address this bias by using measured height, but one's maximum weight in our data is self‐reported. We noted above that the bias in estimates of the relation between obesity and mortality is smaller when a continuous version of BMI is used rather than a set of categories. Accordingly, we use a continuous measure of BMI and thereby expect to reduce but not to eliminate biases associated with misreporting of weight.

5) We address the threats to generalizability associated with restricting analysis to groups that are unusually healthy by including all members of the population, with the exception of one model that is estimated on the set of never‐smokers. The restrictions that have been imposed by many other analysts to reduce reverse causation are largely averted by the use of maximum BMI, which reduces bias from disease‐associated weight loss. By not excluding people with illnesses, we also avoid controlling for any variables that may be on the causal pathway between obesity and death (U4).

## Data and methods

Data for this study were obtained from the National Health and Nutrition Examination Survey (NHANES), a high‐quality nationally representative survey of the non‐institutionalized population of the United States. We integrated data from the NHANES III (1988–1994) and continuous NHANES cohorts (1999–2010) and used linkages to mortality status in the National Death Index through 2011 (National Center for Health Statistics 2016). The study sample included 13,134 adults aged 50–74 at the time of survey with non‐missing data on body mass index, mortality status, and key covariates. There were 2,827 deaths over 120,678 person‐years of follow‐up. Individuals were censored upon reaching age 85.

We used lifetime maximum BMI as the principal indicator of overweight/obesity status in these illustrations. Maximum BMI was calculated by combining data on self‐reported lifetime maximum weight, excluding weight during pregnancy, with data on height measured at survey. We also calculated BMI at the time of survey by combining information on measured height and weight at survey.

We estimated Cox models relating continuous BMI to all‐cause mortality, with age as the underlying time scale. We included a second‐degree polynomial term in the model to capture potential non‐linearity in the association between BMI and mortality (Kivimäki et al. 2008). Our preferred model used maximum BMI as the indicator of overweight/obesity. The model was adjusted for sex, age, race/ethnicity (non‐Hispanic white, non‐Hispanic black, Hispanic, Other), educational attainment (less than high school graduate, high school graduate, and more than high school), and smoking status. For smoking status, individuals were grouped into the categories never, former, and current smokers. Never‐smokers were defined as individuals who smoked fewer than 100 cigarettes during their lifetime. Former and current smokers were further divided based on their intensity of smoking, using the categories less than 1, 1–2, or 2 or more packs per day. Smoking intensity for former smokers was derived from a question that inquired about the number of cigarettes smoked at the time of quitting.

Each of the alternative models modifies the preferred model in a particular way. Departures from the preferred model are not cumulative but rather are considered one at a time. Model 2 uses BMI at survey rather than maximum BMI. Model 3 includes no adjustment for smoking status. Model 4 does not adjust for socioeconomic status (i.e., educational attainment and race are excluded). Model 5 includes whether an individual has been diagnosed with diabetes or “borderline” diabetes, a variable on the causal pathway from obesity to death. Finally, Model 6 is the preferred model but with restriction of the sample to never‐smokers.

We calculated the proportion of deaths attributable to overweight and obesity (population‐attributable fraction (PAF)) using the following formula:

(1) $$PAF\;=\sum_{i=0}^{k}pd_{i}\left(\frac{HR_{i}-1}{HR_{i}}\right)\;$$

where *pd_i_* refers to the proportion of decedents in BMI category I, and *HR_i_* refers to the hazard ratio with respect to mortality for an individual in category *i*. PAF values were calculated using 22.5 kg/m^2^ as the minimum risk value corresponding to a hazard ratio of 1.00 unless the observed nadir was greater than 22.5 kg/m^2^, in which case a hazard of 1.00 was assigned to the higher BMI value. The nadir values were 22.5, 27.5, 24.5, 22.5, 23.5, and 22.5 for Models 1–6, respectively. All BMI values at or below the nadir were assigned a hazard ratio of 1.00 since our goal was to estimate the consequences of excess weight rather than of underweight. So the most precise description of the PAF value resulting from our application of Equation (1) is “the proportion of deaths attributable to above‐optimal body mass index.” We use the term “obesity” in place of “above‐optimal body mass index.” Equation (1) was implemented using the distribution of deaths in single‐BMI units.

The proportional hazards assumption was confirmed by testing the slope of the Schoenfeld residuals by BMI category. We adjusted for unequal probabilities of selection and non‐response using sample weights and accounted for the complex survey design of NHANES. Analyses were performed using STATA 13 (StataCorp, Texas). We estimated variances with the SVY routine, which uses Taylor series linearization.

## Results

Table 1 shows characteristics of the sample population, including mean values of BMI at survey and lifetime maximum BMI. Table 2 presents estimated coefficients of the relationship between BMI and all‐cause mortality for Model 1, the preferred model, and for the five alternative models described above. The quadratic term was positive and statistically significant in Models 1–5, implying that the risks associated with excess weight grow at a faster rate as BMI increases. In contrast, the quadratic term in Model 6, limited to never‐smokers, was slightly below zero and not significant.

**Table 1:** Characteristics of the sample population, NHANES 1988–2010

	No. (n = 13,134)	Percent or mean
Age at survey, years	13,134	60.6
Female	6,476	51.8
Education		
Less than high school	4,744	24.2
High school or equiv.	3,349	30.0
More than high school	5,041	45.8
Race/ethnicity		
Non‐Hispanic white	6,839	80.4
Non‐Hispanic black	2,760	8.7
Hispanic	3,162	7.0
Other	373	3.9
Smoking status		
Never‐smoker	6,231	45.3
Former smoker (packs/day)		
0 to <1	2,186	14.7
1 to <2	1,768	14.8
>2	954	8.8
Current smoker (packs/day)		
0 to <1	920	5.9
1 to <2	858	8.1
>2	217	2.6
BMI, survey (kg/m^2^)	13,134	28.1
BMI, lifetime maximum (kg/m^2^)	13,134	30.3

**Table 2:** Coefficients relating BMI and BMI‐squared to all‐cause mortality

	BMI			BMI‐squared		
**Model**	**Coefficient**	**SE**	**p value**	**Coefficient**	**SE**	**p value**
1	–0.0003	0.013	0.984	0.0021	0.001	0.002
2	–0.0391	0.010	0.000	0.0037	0.001	0.000
3	–0.0096	0.014	0.497	0.0021	0.001	0.003
4	0.0049	0.013	0.708	0.0021	0.001	0.002
5	–0.0053	0.013	0.691	0.0018	0.001	0.008
6	0.0649	0.027	0.018	–0.0005	0.001	0.642

BMI: body mass index; SE: standard error. Model 1: maximum BMI, all smoking groups; Model 2: survey BMI, all smoking groups; Model 3: preferred model without adjustment for smoking status; Model 4: preferred model without adjustment for socioeconomic status; Model 5: preferred model with adjustment for causal pathway variable (diabetes status); Model 6: preferred model with restriction of sample to never‐smokers.

**Figure 1:**
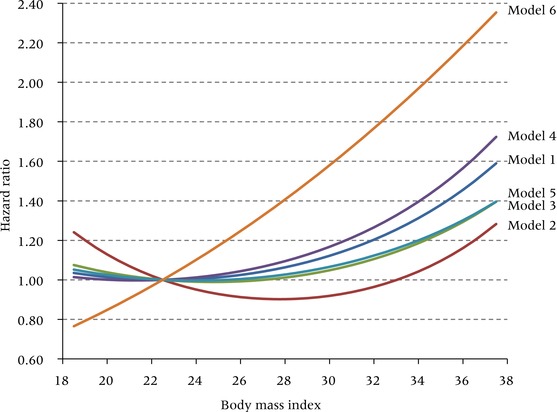
Hazard ratios for all‐cause mortality in one‐unit BMI intervals for six alternative specifications of the model BMI: body mass index. Model 1: maximum BMI, all smoking groups; Model 2: survey BMI, all smoking groups; Model 3: preferred model without adjustment for smoking status; Model 4: preferred model without adjustment for socioeconomic status; Model 5: preferred model with adjustment for causal pathway variable (diabetes status); Model 6: preferred model with restriction of sample to never‐smokers.

Hazard ratios for the various models are presented in Figure 1. For graphical purposes, in each model the hazard ratio at a BMI of 22.5 kg/m^2^ is set at 1.00. In the preferred model, the lowest hazard was observed close to a BMI of 22.5. The curve was essentially flat at lower BMI values and curved upward at higher BMI values. Relative to Model 1, the curve for Model 2 was U‐shaped with hazards increasing in both directions from a nadir of about 27.5 kg/m^2^. Of the remaining models the most significant departure from the pattern was observed in the curve for Model 6, which showed an approximately linear increase in hazards across the entire range of BMI values.

**Figure 2:**
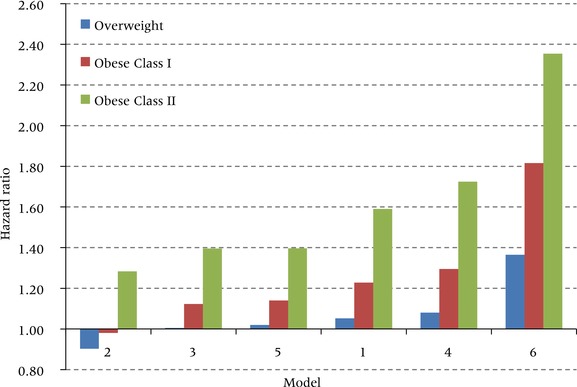
Hazard ratios for all‐cause mortality at the midpoint values of overweight, obese class I, and obese class II for six alternative specifications of the model Midpoint values are 27.5 kg/m^2^ for overweight (25.0‐29.0), 32.5 for obese class I (30.0–34.9), and 37.5 for obese class II (35.0–39.9). Model 1: maximum BMI, all smoking groups; Model 2: survey BMI, all smoking groups; Model 3: preferred model without adjustment for smoking status; Model 4: preferred model without adjustment for socioeconomic status; Model 5: preferred model with adjustment for causal pathway variable (diabetes status); Model 6: preferred model with restriction of sample to never‐smokers.

Figure 2 shows the predicted value of the relative risk for people in the middle of the overweight, obese I, and obese II categories. Model 1, our preferred model, produces a modest estimated risk of death for someone in the middle of the overweight range (BMI of 27.5). Such a person is expected to have 5 percent higher mortality than someone of BMI 22.5. Risks of death rise rapidly thereafter. An individual in the obese I category has a 23 percent higher death rate, while the risk for a person in the obese II category rises to 59 percent. This non‐linearity in relative risks for Model 1 is clearly revealed in Figure 1.

Model 2 shows that the hazards of being overweight or obese are much lower when assessed using weight at survey rather than lifetime maximum weight. Based on weight at survey, overweight is actually protective (hazard ratio of 0.90 compared to a BMI of 22.5) and obese I carries no excess risk (HR = 0.98). These values are similar to those in the Flegal et al. (2013) meta‐analysis, which also used weight at survey. Figure 2 shows that the relative risks associated with each of the excess weight categories estimated using Model 2 are much lower than those produced by any of the other models. Thus, conventional analyses that examine the mortality hazards associated with obesity using weights at survey appear to underestimate those hazards relative to models using maximum BMI.

We next consider in Model 3 the effect on estimated risk when smoking is not controlled. Because smokers have lower average BMI than non‐smokers and are at much higher risk of death, we predicted that failure to control for smoking will induce a downward bias in estimates of mortality associated with obesity. Figure 2 confirms that such a bias occurs: the excess risk associated with being obese II is 1.59 in the preferred model but only 1.40 when smoking is not controlled.

The remaining modifications also confirm the anticipated biases. When no controls are instituted for educational attainment and race/ethnicity, the estimated risk associated with being obese II rises from 1.59 to 1.72. So in Model 4, some of the variance in mortality associated with low socioeconomic status is incorrectly attributed to obesity, with which it is positively associated. The mortality risks of obesity are overestimated when these confounding variables are omitted, as they commonly are.

We use diabetes as an example of a condition on the causal pathway between obesity and death. The lifetime risk of developing diabetes for someone of normal weight is 18.5 percent and for someone who is obese II it is 75.4 percent (Narayan and Boyle 2007). And the risk of dying for someone with diabetes is about 50–90 percent greater than for someone without (Gregg et al. 2012). Diabetes is thus a major disease entity on the causal pathway from obesity to death. When a simple control on diabetes status is added to our preferred model, the relative risk associated with obese II declines from 1.59 to 1.40. At obese I, it declines from 23 percent to 14 percent. So the excess risk declines by about a third in both cases. Controlling one of the major intervening variables between obesity and mortality inappropriately blocks one of the most important pathways through which obesity operates.

Finally, Model 6 applies our preferred model to people who never smoked. By far the highest relative risks are produced when this restriction is imposed. A never‐smoker in the obese II category is predicted to have a death rate that is 2.35 times higher than that of a never‐smoker with a BMI of 22.5. Obesity appears to be a much bigger threat to survival once the competing risks associated with smoking are removed. Although the parameter estimates obtained from Model 6 may be an accurate representation of the risks among never‐smokers, these estimates are not generalizable to the entire population.

**Figure 3:**
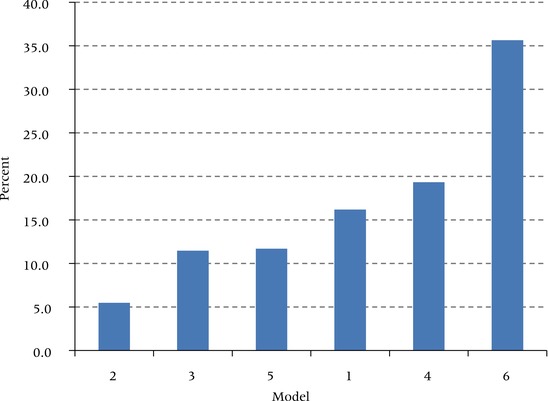
Proportions of deaths at ages 50–84 attributable to overweight or obesity (PAF) for six alternative specifications of the model Model 1: maximum BMI, all smoking groups; Model 2: survey BMI, all smoking groups; Model 3: preferred model without adjustment for smoking status; Model 4: preferred model without adjustment for socioeconomic status; Model 5: preferred model with adjustment for causal pathway variable (diabetes status); Model 6: preferred model with restriction of sample to never‐smokers.

Differences in relative risks produced by the alternative models translate into large differences in the proportion of deaths in this age range attributable to excess weight. Figure 3 presents the population‐attributable fraction (PAF) with respect to all‐cause mortality associated with the six models. Model 1, the preferred model, produces a proportion of deaths in the age interval 50–84 of 16.2 percent. This is a high figure relative to previous estimates (Flegal, Panagiotou, and Graubard 2015). The difference is likely to be primarily a result of our use of maximum BMI, rather than BMI at survey, to represent adiposity. Figure 3 shows that the model using BMI at survey (Model 2) produces a PAF value of only 5.5 percent.

The variation in PAF associated with the other models is consistent with the level of their relative risks. When socioeconomic status is not controlled, PAF rises to 19.3 percent. Placing diabetes on the causal pathway between obesity and death reduces the PAF to 11.7 percent. When smoking is not controlled, the PAF declines to 11.5 percent. Smoking is clearly a major disruptive force in estimating the mortality risks associated with obesity—a conclusion underscored by the very high PAF of 35.6 percent for those who never smoked. That estimate would represent a severe overestimate if assumed to apply to the population as a whole (Flegal 2014).

## Discussion

Estimates of the excess mortality associated with obesity vary sharply across studies. Much of this variation is attributable to differences in model specifications and the imposition of exclusion criteria that compromise generalizability. We have used a high‐quality, nationally representative data set to investigate the biases and threats to generalizability commonly encountered in studies of the mortality risks of obesity.

While we have considered a number of commonly encountered biases, the list is not exhaustive. For example, we have not considered variables such as physical activity, diet, and disability that may be on the causal chain producing obesity and that may themselves have independent effects on mortality (Hernan and Taubman 2008). Diet is difficult to measure both in volume and composition (Kipnis et al. 2003), while the level of physical activity and disability may be results of, as well as causes of, obesity. Nor have we considered disease as a factor in weight determination apart from an effort to circumvent the biases that its actions may introduce. Finally, one of the biases we consider—measurement error in weight—is only partially addressed here. We have reduced the effect of mismeasurement by adopting a continuous functional form for the basic relation, but we are not able to eliminate it by using direct measures of weight.

We have described and attempted to justify our preferred model for estimating the mortality risks associated with obesity. By introducing variations on this model, we have demonstrated dramatic differences in estimates of the magnitude of those risks. Relative to the preferred model, overestimates of the impact on mortality for the population as a whole are produced by failure to control for an individual's socioeconomic status and, especially, by limiting analysis to those who never smoked. Underestimates of mortality risks are produced by controlling variables on the causal pathway between obesity and mortality, by failing to control for smoking, and especially by using survey weight rather than maximum lifetime weight.

The estimated relative risks produced by the various models for someone in the obese II category range from 1.28 (using survey rather than maximum weight) to 2.35 (limited to never‐smokers). The proportion of deaths attributable to obesity for these two models ranges from 5.5 percent to 35.6 percent. These two models represent two very common research strategies. The large majority of studies of the mortality hazards associated with obesity use weights measured at survey, a strategy that produces estimates of those hazards that are much lower than those of any other model specification using maximum weight. We believe that the principal reason for the underestimation is that estimates using survey weight are especially vulnerable to reverse causality, that is, to bias introduced by weight loss among people suffering from illness. In related work, we have shown that as a group, individuals who lose weight from a maximum in the obese range have a higher prevalence of cardiovascular disease and diabetes than those who remain in that class (Stokes and Preston 2016b). However, introducing maximum weight is also expected to reduce biases from using a narrow observational window. Because these biases are addressed simultaneously, we are not able to assess which is more consequential.

Many analyses limit the population under study to those who have never smoked. This limitation does not produce a bias but rather an erroneous impression of hazards for the population as a whole because it eliminates an important competing risk. An alternative interpretation of the high obesity hazard for never‐smokers is that the inclusion of smokers in each of the other five models has induced substantial residual confounding in those models. Some authors assert that, because of residual confounding by smoking, the true association between obesity and mortality can only be observed among never‐smokers (Patel, Hildebrand, and Gapstur 2014; Berrington de Gonzalez et al. 2010). However, such a restriction means that relative risks can never be generalized to the entire population.

The huge differences in relative risks between our preferred model, a model with smoking uncontrolled, and a model estimated on the population of never‐smokers throws the spotlight on smoking. The influence of smoking on mortality in developed countries is profound (Bongaarts 2014), and that influence clearly extends to assessing the effects of other risk factors. We know of no reason to think that the hazards for never‐smokers presented here are themselves biased. This issue is significant because the population of never‐smokers has been growing steadily. In total, 49.9 percent of the US population aged 18+ had never smoked in 1990, a figure that had risen to 62.7 percent by 2015 (Clarke et al. 2015). There is every reason to expect this proportion to continue to grow. Ironically, that favorable development portends an increased hazard and loss of life associated with obesity, highlighting the urgent need for population‐wide campaigns aimed at obesity prevention.
